# John Cheyne

**Published:** 1836-04

**Authors:** 


### JOHN CHEYNE, M.D., F.R.S., M.R.I.A.

*Physician-General to the Forces in Ireland, &c. &c.*

It is with sincere regret we have to record, in this early stage of our labours, the premature death of this very eminent person, at the age of 57. Dr. Cheyne was justly regarded by the whole medical profession in this country as one of the most distinguished and estimable of its members, whether viewed in his professional, literary, or private character. He was the author of numerous works on practical subjects of great value, of which the following are the chief: I. SEPARATE PUBLICATIONS: Disputatio de Rhachitide (Thesis), *Edin.* 1795; Essays on the Diseases of Children, with cases and dissections,—1. on Croup, 2. on Bowel Complaints, particularly the Atrophia Ablactatorum, *Edin.* 1801; an Essay on Hydrocephalus Acutus, or Dropsy in the brain, *Edin.* 1808; the Pathology of the Membrane of the Larynx and Bronchia, *Lond.* 1809; Cases of Apoplexy and Lethargy, with observations upon the comatose diseases, *Lond.* 1812; a second Essay on Hydrocephalus Acutus, *Dubl.* 1815; Essays on Hydrocephalus Acutus, or Water in the Brain, second edition (improved), *Dubl.* 1809; an account of the Fever lately epidemic in Ireland (in conjunction with Dr. F. Barker), two vols. *Dubl.* 1821.—II. EDINBURGH MEDICAL JOURNAL: Observations on the Effects of Purgatives, iv. 310, 1808; Case of Bronchial Polypus, with introductory observations, iv. 441, 1808.—III. DUBLIN HOSPITAL REPORTS: Vol. i. 1818: Report of the Hardwicke Fever Hospital for the year ending on the 31st March, 1817; a case of Melæna, with observations on the alternate excess of morbid action in the mucous and serous membranes; on the virtues of James's Powder in the Apoplectic Diathesis. Vol. ii. 1818: Report of the Hardwick Fever Hospital for the year ending on the 31st March, 1818, including a brief account of an epidemic Fever in Dublin; Case of Apoplexy in which the fleshy part of the heart was converted into fat. Vol. iii. 1822: Report of the Whitworth Hospital, containing an account of Dysentery as it appeared in Dublin in the latter end of 1818, with a brief account of the disease as it appeared in Limerick in 1821. Vol. iv. 1827: Medical Report on the feigned Diseases of Soldiers; Cases of a fatal Erethism of the Stomach. Vol. v. 1830: Small and frequently repeated Bleedings in Hæmoptysis and Incipient Phthisis, recommended, &c.—IV. CYCLOPÆDIA OF PRACTICAL MEDICINE: vol. i. 1832, Croup; vol. ii. 1832, Epilepsy, Epidemic Gastric Fever; vol. iii. 1833, Laryngitis; vol. iv. 1834, Wakefulness.

For the following few particulars of the life and character of Dr. Cheyne, we are indebted to the pages of two of our contemporaries; and as they are at once creditable to them and suited to our purpose, we make no apology for extracting them.

"Dr. J. Cheyne was born on the 3d of February, 1777. He was originally of Leith. After having been some years Surgeon in the Artillery Corps, he returned to Leith, where he obtained considerable practice. In 1795 he took the degree of M.D. at Edinburgh. Having married an Irish lady he was induced, about the year 1809, to settle in Dublin, where he obtained the appointment of Professor of Medicine, and became Physician to the Meath Hospital. The confidence of the profession in his experience and excellent judgment, with his delicate and honorable conduct, drew him into the highest and most extensive practice, and obliged him to resign his public duties, except those belonging to the important post of Physician-General, to which he was appointed in the year

1820. For many years his professional receipts were very large; but his health broke under the fatigue. When in a state of great debility, a domestic calamity occurred, which seemed quite to overwhelm him: one of his sons was accidentally shot while on an excursion in the country, and unfortunately, as Dr. Cheyne hurried from town to his assistance, the postilion rode over and killed a child. These distressing circumstances, we have reason to believe, hastened his retirement from Dublin; his departure from which metropolis drew forth public expressions of regret from all the medical bodies. He brought his family with him to England, and bought a property in Buckinghamshire, where he lived in retirement during the last few years. He died, after an illness of some months, on the 31st of January, 1836."—*Med. Gazette, No.* 430.

"No man ever maintained, in the circle in which he practised, more respect and confidence from his professional brethren, or a higher character with the public as a skilful physician. In consultation, he displayed a penetration in the diagnosis of disease, and a readiness and sincerity in the communication of his experience in similar cases, which never failed to secure the confidence of the practitioner at whose recommendation he had been called to the attendance. He had, moreover, the enviable quality of observing the most honorable conduct toward the gentleman in attendance with him, without a compromise of the duty which he owed to the invalid: he directed the treatment, without arrogating to himself any merit for its success; and assisted the efforts of his junior, without lessening towards him the confidence of his patient. Dr. Cheyne's punctuality to appointments was another feature in his character which rendered professional intercourse with him peculiarly satisfactory; and even in the days of his most varied and extensive practice, he treated the youngest member of the profession with the same polite consideration in this particular as the oldest. Such was the estimation in which he was held, and such the universal regret at his retirement from practice, that addresses were presented to him from three different branches of the profession requesting him to return and resume his station amongst them; a compliment to an individual unprecedented in the annals of medicine."—*Dublin Journal, No.* 25.

